# Extract from the *Coriolus versicolor* Fungus as an Anti-Inflammatory Agent with Cytotoxic Properties against Endothelial Cells and Breast Cancer Cells

**DOI:** 10.3390/ijms21239063

**Published:** 2020-11-28

**Authors:** Tomasz Jędrzejewski, Justyna Sobocińska, Małgorzata Pawlikowska, Artur Dzialuk, Sylwia Wrotek

**Affiliations:** 1 Department of Immunology, Faculty of Biological and Veterinary Sciences, Nicolaus Copernicus University, 1 Lwowska Str., 87-100 Torun, Poland; j.sobocinska@umk.pl (J.S.); m_pawlikowska@umk.pl (M.P.); wrotek@umk.pl (S.W.); 2 Department of Genetics, Faculty of Biological Sciences, Kazimierz Wielki University, 10 Powstańców Wielkopolskich Ave., 85-090 Bydgoszcz, Poland

**Keywords:** *Coriolus versicolor* extract, inflammation, lipopolysaccharide, breast cancer cells, endothelial cells, Toll-like receptor 4, phosphorylated IκB

## Abstract

Chronic inflammation is a well-recognised tumour-enabling component, which includes bioactive molecules from cells infiltrating the tumour microenvironment and increases the risk of cancer progression. Since long-term use of the currently available anti-inflammatory drugs used in cancer therapy causes numerous side effects, the aim of this study was to investigate the effect of an extract isolated from the *Coriolus versicolor* fungus (CV extract) on HUVEC endothelial cells and MCF-7 breast cancer cells in a pro-inflammatory microenvironment mimicked by lipopolysaccharide (LPS). The cells were simultaneously stimulated with the LPS and CV extract. After co-treatment, the cell viability, generation of reactive oxygen species (ROS), wound-healing assay, production of the pro-inflammatory and pro-angiogenic factors (interleukin (IL) 6, IL-8, and metalloproteinase (MMP) 9)), as well as expression of Toll-like receptor (TLR) 4 and phosphorylated IκB (p-IκB) were evaluated. The results showed that the CV extract inhibited IL-6, IL-8, and MMP-9 production by the LPS-stimulated cells. This effect was accompanied by a decrease in TLR4 and p-IκB expression. The CV extract also had anti-migratory properties and induced a cytotoxic effect on the cells that was enhanced in the presence of LPS. The observed cytotoxicity was associated with an increase in ROS generation. We conclude that the CV extract possesses cytotoxic activity against cancer cells and endothelial cells and has the ability to inhibit the expression of the pro-tumorigenic factors associated with inflammation.

## 1. Introduction

An extract from the *Coriolus versicolor* (CV extract) fungus has been classified as a biological response modifier (BRM) with potential therapeutic application [1]. The results of numerous experimental studies and clinical trials demonstrate that the CV extract inhibits the growth of cancer cells [2,3,4,5]. It is well known that the anti-tumour properties of the CV extract are mediated not only through direct cytotoxic effects, but also by immunomodulating regulation. The extract from *Coriolus versicolor* increases the activity of monocytes and natural killer cells, the production of reactive oxygen species (ROS) [6,7,8,9], and the production of cytokines, such as tumour necrosis factor (TNF) α, interferon (IFN) γ, or interleukin (IL)-6. Importantly, the CV extract is clinically well tolerated and do not show any toxic effect, even in high doses and with long-term use [1,5].

Chronic inflammation is a well-recognised tumour-enabling capability, which can promote cancer development and allows nascent tumours to escape immunosurveillance [10]. Inflammation can increase the risk of cancer progression by providing bioactive molecules from the inflammatory cells infiltrating the tumour microenvironment. These include cytokines such as TNF-α, IL-6, or IL-8, among others, which maintain a sustained proliferative rate either by promoting cell proliferation and angiogenesis or by inhibiting apoptosis. An inflammatory state also activates extracellular matrix-modifying enzymes, such as metalloproteinases (MMP), which promote the epithelial–mesenchymal transition and facilitate other carcinogenesis programs, such as genome instability, reprogramming of energy metabolism, and immune evasion [11,12]. Moreover, in response to inflammatory agents, endothelial cells are the main cellular player in the secretion of pro-angiogenic factors, such as vascular endothelial growth factor (VEGF) and IL-8 [13].

Recent advances in cancer immunotherapy, particularly immune checkpoint blockade therapy, have dramatically changed the therapeutic strategy against advanced malignancies. Growing evidence suggests that the major barrier to more successful cancer immunotherapy is the tumour microenvironment, where chronic inflammation has a predominant role in tumour survival, angiogenesis, and immunosuppression. Over the past decades, the understanding of cancer-related inflammation has significantly evolved, and now various therapeutic options tailored to the tumour microenvironment exist [14]. These therapeutic strategies include, among others, the application of monoclonal antibodies that are targeting immune checkpoint inhibitors associated with inflammation and T cell activity, such as cytotoxic T lymphocyte antigen-4 (CTLA-4) and programmed death-1 (PD-1). These antibodies restore the active anti-tumoral immune response [15]. Moreover, over the past few years, several other novel targeted therapies have been approved for cancer treatment, including drugs with new targets (i.e., CDK4/6 inhibitors), mutation targeting drugs (i.e., the EGFR T790M targeting osimertinib), drugs with multiple targets (i.e., the EGFR/HER2 dual inhibitor neratinib), and drug combinations (i.e., encorafenib/binimetinib and dabrafenib/trametinib). All these therapies usually present with high selectivity, precisely targets a specific gene or protein, and exert a biological function with minimal side effects, which has distinguished them from most conventional non-specific chemotherapeutic drugs [16].

Unfortunately, not all the studies confirm the great efficiency of targeted therapy regarding the cancer patients with chronic inflammation [17]. Therefore, several anti-inflammatory drugs (nonsteroidal anti-inflammatory drugs and corticosteroids) that are able to reduce the mortality of patients with certain types of cancer are still in use [18,19]. However, the long-term use of these drugs can result in side effects, including renal failure, intestinal inflammation, and adrenal suppression, among others [20,21]. Accordingly, new anti-inflammatory agents are currently being sought. Since we have previously shown that the CV extract attenuates lipopolysaccharide (LPS)-induced synthesis of pro-inflammatory cytokines by peripheral blood mononuclear cells (PBMCs) [9], the aim of the present study was to examine the effect of this extract on human umbilical vein endothelial cells (HUVECs) and the MCF-7 breast cancer cell line in a pro-inflammatory microenvironment mimicked by LPS. It is well established that during inflammation endothelial cells as well as cancer cells release numerous factors, which provide a favourable microenvironment for the growth of tumours [13,22]. Moreover, since it is known that LPS activates the TLR4-mediated signalling pathway [23], and the phosphorylation of IκB at Ser32 and Ser36 induces the release and nuclear translocation of active nuclear factor κB (NF-κB) [24], leading to pro-inflammatory gene expression, in the present study we also evaluated the expression of TLR4 and p-IκB in the cells co-treated with the CV extract and LPS. Our results showed that it decreased the release of pro-inflammatory and pro-angiogenic cytokines (IL-6 and IL-8) and MMP-9, which are regarded as factors for breast cancer cell invasion and stimulation of angiogenesis. This effect was accompanied by a decreased expression of TLR4 and p-IκB. We also observed the anti-migratory properties and cytotoxic effect of the CV extract on the HUVEC and MCF-7 cells, which were enhanced in the pro-inflammatory microenvironment. This cytotoxicity was associated with an increase of intracellular ROS generation.

## 2. Results

### 2.1. LPS Stimulation Enhances the Cytotoxicity of the CV Extract against HUVEC and MCF-7 Cells

**Figure 1:**
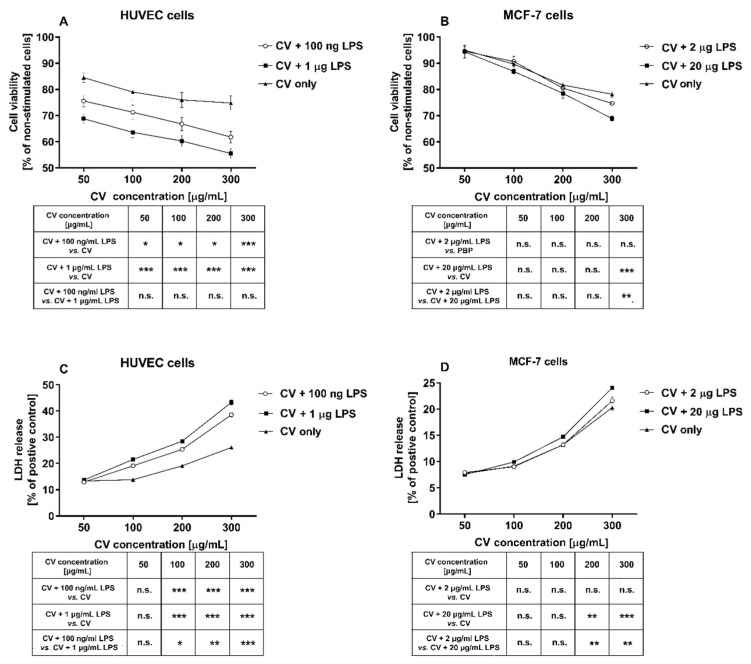
Cell viability of human umbilical vein endothelial cells (HUVECs) (**A**) and MCF-7 human breast cancer cells (**B**) co-stimulated with different concentrations of the *Coriolus versicolor* extract (CV), in the presence or absence of lipopolysaccharide (LPS). Cell viability was assessed using the MTT assay. The results are presented as the percentage of non-stimulated control cells (served as 100%). (**C**) and (**D**) show the level of lactate dehydrogenase (LDH) released from the cells treated with CV and LPS. The results are expressed as the percentage of the positive control cells treated with 1% Triton X-100 solution (served as 100%). The data are shown as the means ± standard error of three independent experiments with six wells in each experiment. The tables below the graphs present significant differences between the CV-treated cells compared with the cells co-stimulated with CV and LPS (* *p* < 0.05; ** *p* < 0.01; *** *p* < 0.001; n.s. indicates non-significance (*p* > 0.05)).

The cytotoxic effect of the CV extract on the LPS-stimulated HUVEC endothelial cells and MCF-7 breast cancer cells was assessed using two different assays. Cell viability was measured using the MTT reagent, whereas the level of cell death was determined by measuring the lactate dehydrogenase activity (LDH assay). Cell viability was significantly decreased following the CV extract challenge alone in a dose-dependent manner in both the HUVEC and MCF-7 cells (by 75% and 78%, respectively; Figure 1A,B). Importantly, the viability of the HUVEC cells co-stimulated with the CV extract and LPS was significantly lower when compared to cells treated with the CV extract alone. This phenomenon was observed for both doses of LPS, with the cell viability decreasing by 60% with the 100 ng/mL dose and 55% for the 1 µg/mL dose (*p* < 0.001). In contrast, the difference in viability of the MCF-7 cells between the CV extract-stimulated cells and the cells co-treated with the CV extract and LPS was observed only for the highest concentration of CV extract (300 µg/mL) and a higher dose of LPS (20 µg/mL) (*p* < 0.001). None of the LPS doses used alone had an effect on cell viability.

The LDH release assay confirmed the results of the MTT assay. As compared with the positive control, the CV extract-stimulated HUVEC cells and MCF-7 cells exhibited LDH release in a dose-dependent manner. Moreover, co-treatment of the HUVEC cells with LPS and the CV extract induced a significantly higher LDH release compared with the cells stimulated with the CV extract only. This effect was observed for CV extract concentrations from 100 to 300 µg/mL (*p* < 0.001; Figure 1C). On the other hand, the MCF-7 cells simultaneously exposed to the CV extract and LPS released a higher amount of LDH in comparison with the CV extract-treated cells, but only when were treated with a higher concentration of LPS (20 µg/mL) and the two highest doses of CV extract (200 and 300 µg/mL, *p* < 0.01 and *p* < 0.001, respectively; Figure 1D). Exposure to LPS alone did not increase the LDH leakage in the HUVEC and MCF-7 cells, compared with the controls.

Using data from the MTT and LDH assays, the half inhibitory concentration (IC_50_) was estimated (Table 1). IC_50_ is the concentration of compounds that causes a 50% reduction in viable cells (MTT assay) or a 50% release of LDH (LDH assay). Values for IC_50_ obtained by the MTT assay were higher than those obtained by the LDH test. However, all IC_50_ results clearly indicate that co-treatment of HUVEC and MCF-7 cells with the CV extract and LPS was more cytotoxic in terms of metabolic activity and membrane integrity than stimulation of the cells with the CV extract only.

### 2.2. LPS Stimulation Increases ROS Level in the CV Extract-Treated Cells

**Figure 2:**
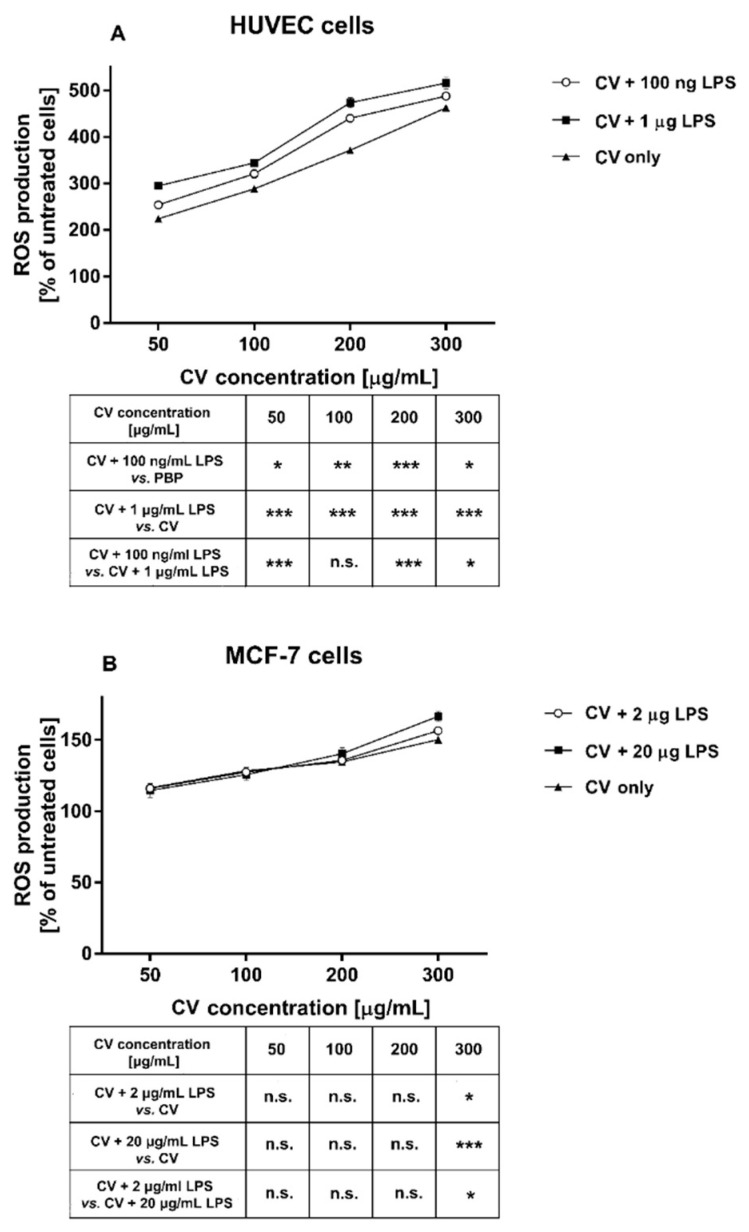
The level of reactive oxygen species (ROS) in human umbilical vein endothelial cells (HUVECs) (**A**) and MCF-7 human breast cancer cells (**B**) co-treated with different concentrations of the extract from *Coriolus versicolor* (CV), with or without different doses of lipopolysaccharide (LPS). Intracellular production of ROS was determined using DCFH-DA. Data show the mean ± SEM of three independent experiments with five wells in each experiment. The results are presented as a percentage of the untreated control cells (served as 100%). The tables below the graphs present significant differences between the CV extract-stimulated cells and the cells co-treated with the CV extract and LPS (* *p* < 0.05; ** *p* < 0.01; *** *p* < 0.001; n.s. indicates non-significance (*p* > 0.05)).

To assess the effect of the co-treatment of the cells with the CV extract and LPS on ROS production, the HUVEC and MCF-7 cells were stained with 2′7′-dichlorodihydrofluorescein diacetate. As shown in Figure 2, the CV extract treatment increased ROS generation in both cell lines in a dose-dependent manner. However, the HUVEC cells co-stimulated with the CV extract and LPS released a higher amount of ROS than the cells treated with the CV extract only, and this effect was observed for all doses of CV extract used (Figure 2A). The MCF-7 cells were less sensitive to simultaneous stimulation with the CV extract and LPS. The level of ROS in these cells was higher only when the cells were stimulated with the highest concentration of CV extract (300 µg/mL; Figure 2B). The stimulation of cancer cells with LPS did not affect the level of ROS generation, whereas the LPS treatment of the HUVEC cells slightly increased ROS generation (by 24% and 151% for the LPS doses of 100 ng/mL and 1 µg/mL, respectively; *p* < 0.05).

### 2.3. Anti-Migratory Activity of the CV Extract

**Figure 3:**
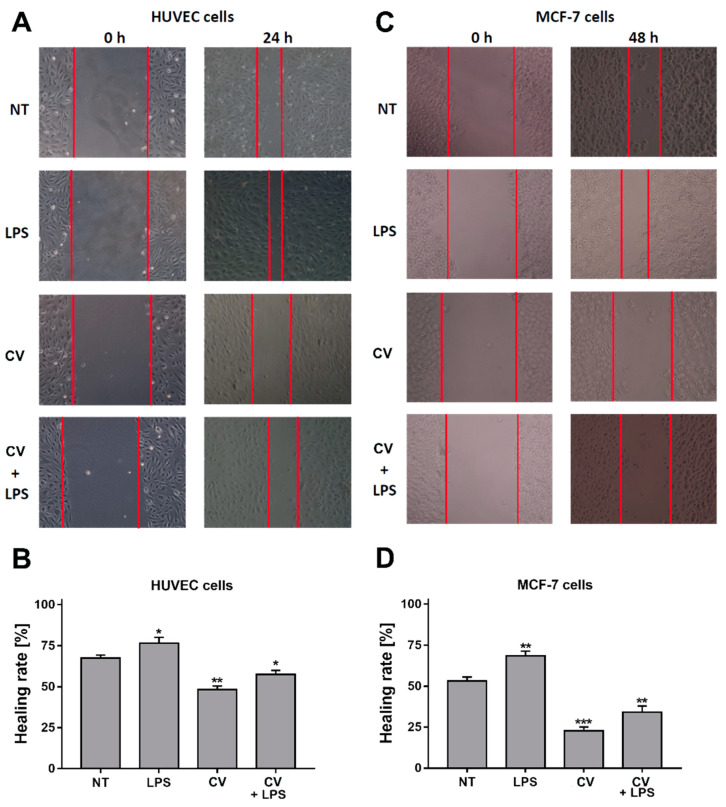
Anti-migratory activity of the extract from *Coriolus versicolor* (CV) on the human umbilical vein endothelial cells (HUVECs) (**A**,**B**) and MCF-7 human breast cancer cells (**C**,**D**). HUVECs were stimulated with the CV extract (100 µg/mL) and/or LPS (100 ng/mL) for 24 h, and the MCF-7 cells were treated with the CV extract (100 µg/mL) and/or LPS (20 µg/mL) for 48 h. (**A**) and (**C**) show the representative images of the 0-, 24-, or 48-h-lasting treatment with the compounds (magnification: × 40; scale bar: 50 μm). (**B**) and (**D**) present the quantitative closure (%) measured using ImageJ software. The data are shown as the mean ± SEM of three independent experiments, each with three measuring points. The results were expressed as the percentage of the non-treated cells. Asterisks show significant differences between the untreated cells and the cells stimulated with the compounds (* *p* < 0.05, ** *p* < 0.01, *** *p* < 0.001).

To investigate the anti-metastatic activity of the CV extract, a wound healing assay was performed to estimate cell migration. The healing degree of scratches reflected the inhibition effect of the CV extract on the migration ability of the tumour cells and endothelial cells in the pro-inflammatory microenvironment. As shown in Figure 3, the CV extract treatment alone most effectively inhibited migration of the HUVEC cells (48.2 ± 2.1**%*)*** and MCF-7 cells (22.9 ± 2.2%). In contrast, the cells stimulated with LPS demonstrated the highest healing rate (76.5 ± 3.6**%** and 68.4 ± 3.0%, respectively). Importantly, the presence of the CV extract in the LPS-treated media partially inhibited this effect (57.5 ± 2.5**%** and 34.1 ± 3.8%, respectively)

### 2.4. CV Extract Decreases the LPS-Induced Release of IL-6, IL-8, and MMP-9 from Cells

**Figure 4:**
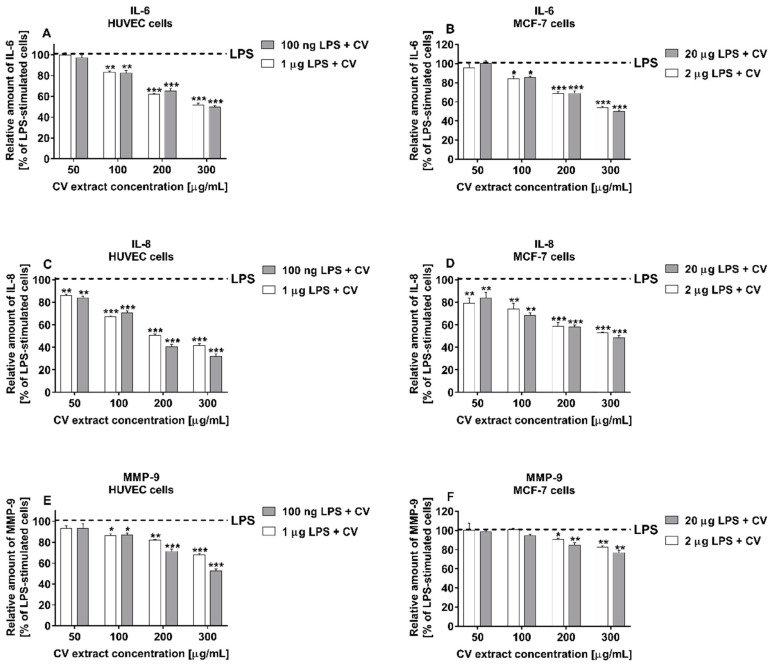
Inhibitory effect of the *Coriolus versicolor* extract (CV) on the LPS-induced release of IL-6 (**A**,**B**), IL-8 (**C**,**D**), and MMP-9 (**E**,**F**) from human umbilical vein endothelial cells (HUVECs) and MCF-7 human breast cancer cells, respectively. Data are shown as the mean ± SEM of five independent experiments. Total levels of the cytokines and MMP-9 in the culture media were measured by ELISA assays and normalised to the total protein amount of the viable cell pellets. The results are expressed as the percentage of LPS-stimulated cells (served as 100% at the respective dose, indicated by dashed lines). Asterisks show significant differences between the cells co-stimulated with the CV extract and LPS in comparison with the cells treated with LPS at the appropriate concentration (* *p* < 0.05; ** *p* < 0.01; *** *p* < 0.001).

IL-6, IL-8, and MMP-9 belong to the group of factors that participate in breast cancer cell invasion and adhesion. As shown in Figure 4, co-treatment of the cells with the CV extract and LPS significantly inhibited the LPS-induced production of these factors in a dose-dependent manner compared with the cells stimulated with LPS only. This effect was observed for both doses of LPS tested. Stimulation of both cell lines with the CV extract at doses of 100–300 µg/mL decreased the LPS-induced production of IL-6 by about 50% for the highest CV extract concentration (Figure 4A,B). The cells co-treated with the CV extract and LPS also produced significantly lower amounts of IL-8. The highest CV extract concentration inhibited the IL-8 secretion from the LPS-treated HUVEC cells by 30–40% and by 50% from the MCF-7 cells (Figure 4C,D, respectively). Finally, the CV extract at doses of 100–300 µg/mL also reduced the production of MMP-9 by the LPS-treated HUVEC cells (by 52% or 68% for cells stimulated with LPS at doses of 100 ng/mL or 1 µg/mL, respectively; Figure 4E). In contrast, the CV extract inhibited the secretion of MMP-9 from the LPS-stimulated MCF-7 cells when the cells were treated with the CV extract at the two highest doses (Figure 4F).

**Figure 5:**
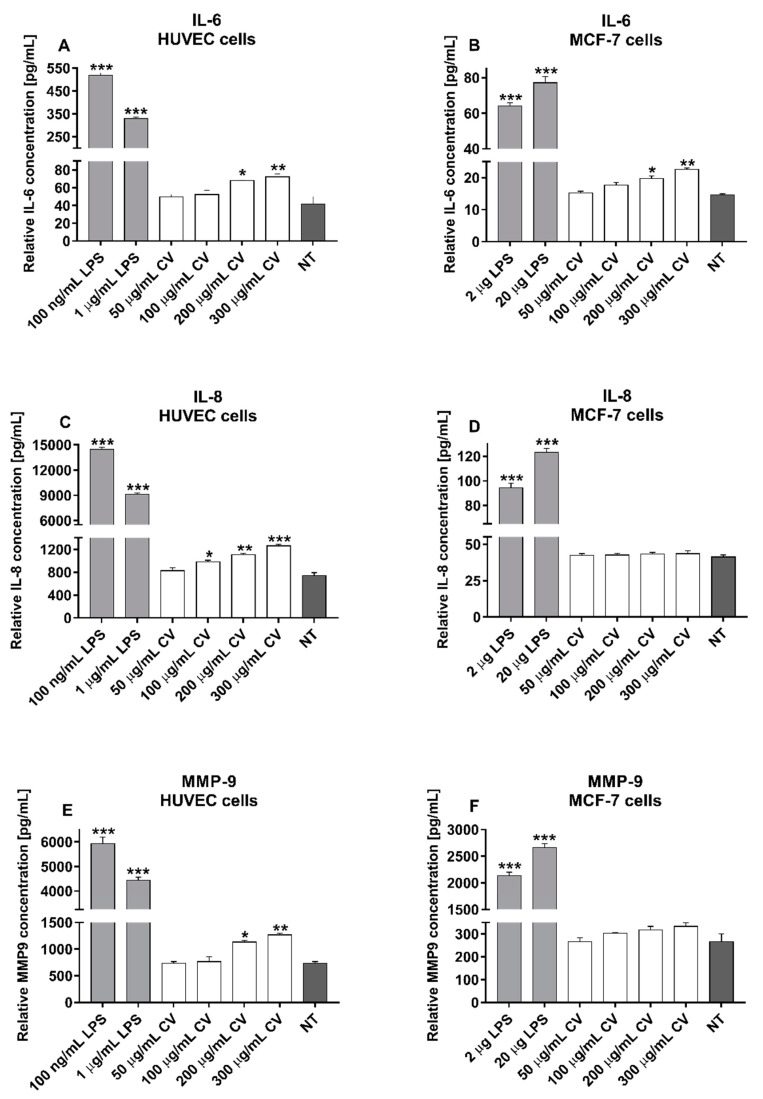
Effect of the extract from *Coriolus versicolor* (CV) on the production of IL-6 (**A**,**B**), IL-8 (**C**,**D**), and MMP-9 (**E**,**F**) by human umbilical vein endothelial cells (HUVECs) and MCF-7 human breast cancer cells. Data are shown as the mean ± SEM of five independent experiments. The concentrations of the cytokines and MMP-9 in the culture media were normalised to the total protein amount of the viable cell pellets. Asterisks indicate significant differences between the cells stimulated with LPS or the CV extract and the untreated cells (* *p* < 0.05; ** *p* < 0.01; *** *p* < 0.001).

Figure 5 shows the concentrations of IL-6, IL-8, and MMP-9 in the culture media collected after stimulation of the HUVEC or MCF-7 cells with the CV extract only. These results were compared with the levels of the cytokines and MMP-9 measured in the supernatants from the LPS-stimulated cells and untreated cells. Compared with the unstimulated HUVEC cells, the CV extract-treated endothelial cells produced significantly higher quantities of IL-6 (Figure 5A), IL-8 (Figure 5C), and MMP-9 (Figure 5E) when the cells were stimulated with the CV extract at doses of 200 and 300 µg/mL. The IL-8 levels were also increased after the CV extract treatment at a dose of 100 µg/mL. In contrast, the CV extract-stimulated MCF-7 cancer cells only released higher levels of IL-6 compared to untreated cells (Figure 5B), while the concentrations of IL-8 and MMP-9 were comparable with the control cells (Figure 5D,F, respectively). Importantly, the levels of IL-6, IL-8, and MMP-9 measured in the culture media collected from the CV extract-treated cells were many times lower than the levels observed in the culture media collected from the LPS-stimulated cells.

### 2.5. CV Extract Decreases LPS-Induced Expression of TLR4 and Phosphorylated IκB

**Figure 6:**
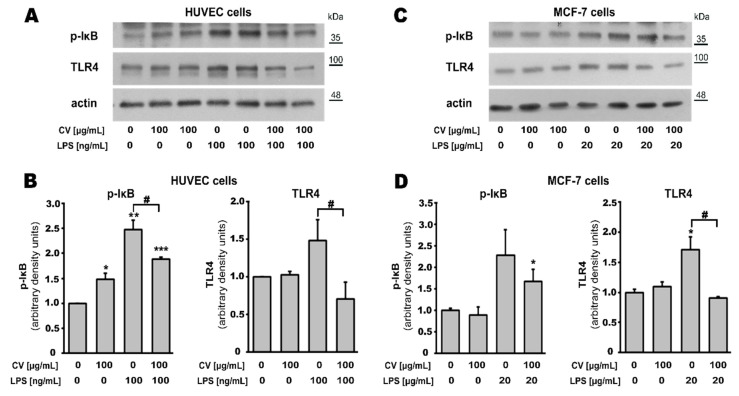
Effect of the extract from *Coriolus versicolor* (CV) and LPS on the p-IκB and TLR4 levels in human umbilical vein endothelial cells (HUVECs) (**A**,**B**) and MCF-7 human breast cancer cells (**C**,**D**). HUVECs were stimulated with the CV extract (100 µg/mL) and/or LPS (100 ng/mL) for 24 h, and the MCF-7 cells were treated with the CV extract (100 µg/mL) and/or LPS (20 µg/mL) for 48 h. Cell lysates were analysed by immunoblotting of phosphorylated IκB (p-IκB) and TLR4 relative to actin content. The blots were quantified by densitometry using ImageJ software (**B**,**D**). The results are expressed as a fold-change compared to the unstimulated control cells (**B**,**D**). Asterisks indicate significant differences between the stimulated cells and untreated cells (* *p* < 0.05; ** *p* < 0.01; *** *p* < 0.001). Hash marks indicate significant difference between the cells stimulated with LPS and the cells co-treated with LPS and the CV extract (^#^ *p* < 0.05).

Results of the Western blot analysis showed that the CV extract decreased the LPS-induced activation of TLR4 both in HUVEC endothelial cells (Figure 6B; *p* < 0.05) and MCF-7 breast cancer cells (Figure 6D; *p* < 0.05). Similarly, the presence of the CV extract reduced the expression of p-IκB in the HUVEC cells stimulated with LPS (*p* < 0.05) and tended to reduce the expression of p-IκB in the LPS-treated MCF-7 cells (*p* = 0.09). Moreover, we also observed that stimulation of the HUVEC cells with the CV extract increased the protein level of p-IκB in comparison with the untreated cells (Figure 6B; *p* < 0.05).

## 3. Discussion

In our study, an extract from *Coriolus versicolor* was isolated from commercially available capsules. The purchased CV powder was obtained by the manufacturer using biofermentation and subsequent processing with the method of hot-water extraction. This type of extraction allows to break the chitin barrier and extract the maximum amount of the bioactive compounds from the mushroom. It is well established that a multistep hot-water extraction of the CV fungus’s biomass appears to be necessary to recover the active polymers in sufficient amounts for use in modern commercial preparations. The best-known commercial protein-bound polysaccharides isolated from the CV fungus are polysaccharopeptide Krestin (PSK) and polysaccharopeptide PSP [4]. Both products have similar physiological activities and they are obtained by batch fermentation. However, PSK is recovered from hot-water extracts of the biomass by salting out with ammonium sulphate, whereas PSP is recovered by alcoholic precipitation from the hot-water extract. There are several other potential approaches to effectively obtain protein-bound polysaccharides from the CV extract, including extraction of the mycelial biomass with aqueous alkaline solution extraction or extraction with hot aqueous solutions containing a surfactant, such as 2% Triton X-100 [5]. Importantly, the variation in the preparation and extraction methods of the protein-bound polysaccharides have a direct influence on the biological activities of the CV extract [25].

The relationship between chronic inflammation and promotion of tumour metastasis is well established. It is considered to be one of the most prominent epigenetic and environmental factors contributing to oncogenesis and tumour progression [10]. Therefore, the use of anti-inflammatory agents is believed to be essential for cancer prevention and therapy. Since long-term use of nonsteroidal anti-inflammatory drugs and others anti-inflammatory drugs, such as corticosteroids can result in serious side effects [20,21], new anti-inflammatory agents are currently being sought. Although we found in our previous study that the CV extract attenuates LPS-induced synthesis of pro-inflammatory cytokines by peripheral blood mononuclear cells [9], the influence and mechanism of action of the CV extract on the cells involved in tumorigenesis have not been investigated yet. Therefore, in the present study we evaluated the effect of the CV extract on MCF-7 breast cancer cells and HUVEC endothelial cells that were co-stimulated with LPS to mimic the chronic inflammatory environment. The results of the MTT and LDH assays showed that the CV extract significantly inhibited the metabolic activity and increased the cytotoxicity in both cell lines. These data confirm the results of other authors and our previous findings, showing that the CV extract possesses anti-proliferative activity against tumour cells, including triple-negative breast cancer cells, amelanotic melanoma cells and liver cancer cell lines [8,26,27], and endothelial cells [28]. However, in our study, the CV extract was toxic to the MCF-7 breast cancer cells when using high doses, which may indicate the ineffectiveness of the CV extract for practical use. Moreover, the IC_50_ values for the cancer and endothelial cells were similar, which may show the potential toxic effect of the CV extract on normal cells. Therefore, the effect of the CV extract on other cancer and normal cell types should be investigated in future experiments to look for cancer cells that will be more sensitive to the CV extract and for normal cells that will be less sensitive to the their action, respectively. Nevertheless, HUVEC cells are a commonly used model system to study angiogenesis *in vitro*. Thus, in the context of tumour angiogenesis, the cytotoxic and anti-migratory effect of the CV extract on these cells, in parallel with the ability of the CV extract to reduce the production of pro-angiogenic factors (IL-6, IL-8 and MMP-9) during chronic inflammation, are seen to be a beneficial phenomenon. However, further studies using co-cultures of endothelial and cancer cells in the inflammatory environment are required to confirm the effectiveness of the CV extract. Surprisingly, in the present study we also demonstrated that co-treatment of HUVEC cells and MCF-7 cells with the CV extract and LPS simultaneously resulted in a greater increase in cytotoxicity in terms of metabolic activity and membrane integrity than stimulation of cells with the CV extract only. The type of cell death observed is unknown and requires further investigation.

In the present paper we also showed that the CV extract displayed anti-migratory activity against both tested cell lines. Moreover, the presence of the CV extract in the culture medium reduced the migration level of the LPS-treated cells. This effect is thought to be beneficial since experimental evidence suggests that following exposure to LPS the migration and invasion of cancer cells and endothelial cells is increased as a result of the stimulation of the TLR4 pathway [29,30].

It is thought that the specific anti-proliferative effect of several fungal extracts, including the CV extract, on cancer cells is associated with oxidative stress [26,31,32]. Here, we documented that intracellular ROS generation in the CV extract-stimulated cells was potentiated in the presence of LPS, which suggests the involvement of ROS in this process. It is well established that excessive oxidative stress can induce programmed cell death [33]. Furthermore, our previous findings showed that the CV extract induces programmed death of cancer cells via ROS generation [3,26]. However, to the best of our knowledge, this is the first report revealing the ROS-dependent cytotoxic activity of the CV extract in the pro-inflammatory environment.

That the inflammatory microenvironment stimulates cancer cells and endothelial cells to secrete pro-inflammatory and pro-angiogenic cytokines and metalloproteinases, which induce cancer cell invasion and tumour angiogenesis, is well known [34,35,36]. Our previous findings and the results of other authors have shown that the CV extract inhibits the LPS-induced production of pro-inflammatory cytokines in immune cells [9,25]. In the present study, we investigated the effect of a CV extract on the production of pro-tumorigenic factors by breast cancer cells and endothelial cells in the pro-inflammatory microenvironment. Our results demonstrated that the CV extract decreased the LPS-induced production of IL-6. This cytokine acts intrinsically on tumour cells through numerous downstream mediators to support cancer cell proliferation, survival, and metastatic dissemination [37]. We also observed a reduction in IL-8 production, which, according to current knowledge, prevents tumour progression through the inhibition of angiogenesis, cancer cell growth and survival, and tumour cell migration [38,39]. Another prognostic indicator of poor outcome in cancer patients is MMP-9, which has widely been found to relate to the pathology of cancers, including tumour growth, invasion, metastasis, and angiogenesis [40]. Our findings clearly indicate that the CV extract is also able to decrease the secretion of this pro-tumorigenic factor. We also demonstrated that, unlike in untreated endothelial cells, the stimulation of these cells with the CV extract increased the production of IL-6, IL-8, and MMP-9, whereas cancer cells only released slightly more IL-6. Our results, along with the findings of other authors, indicate the ability of the CV extract to induce the production of many cytokines, including IL-1β, IL-2, IL-6, IL-10, TNF-α, and IFN-γ, by immune cells and cancer cells [2,7,9,25]. Importantly, the levels of all these factors in the culture media collected from the CV extract-treated cells were many times lower compared with those observed in the culture media from the LPS-stimulated cells. These results indicate that CV extract is a weaker stimulator of expression of genes encoding pro-inflammatory cytokines than LPS.

It is well known that LPS activates the TLR4-mediated signalling pathway, inducing cytokine production [23] and, in a resting cell, the stable IκB inhibits cytoplasm-residing NF-κB p65 in an inactive form. However, LPS stimulates a cascade of effects, including degradation of p-IκB, and leads to the release of NF-κB p65, which translocates into the nucleus to initiate transcription of the pro-inflammatory cytokines [41]. Similarly, several studies showed that the CV extract interacts with immune cells through the TLR4 signalling pathway, and that the expression of cytokines induced by the CV extract as well as by LPS is correlated with its effect on NF-κB transcription [4,42]. Therefore, since we have shown that the co-stimulation of cells with the CV extract and LPS affects the cell viability and cytokine production, in the present study we analysed the effect of the CV extract on the LPS-induced expression of TLR4 and p-IκB. Our results demonstrated that the CV extract decreased the expression of TLR4 in the LPS-stimulated HUVEC cells and MCF-7 cells and also prevented the phosphorylation of IκB in these cells. This effect is thought to be beneficial since experimental evidence suggests that persistent activation of TLR4-induced inflammatory signalling during chronic inflammation can contribute to carcinogenesis [43], and cancer cell survival, proliferation, migration, and invasion are induced by triggering of the TLR4–NF-κB signalling pathway under inflammatory conditions [44,45]. Moreover, a significant number of cancer patients display constitutive NF-κB activity due to the inflammatory microenvironment and various oncogenic mutations. NF-κB activity also promotes tumour cell proliferation, suppresses apoptosis, and induces angiogenesis [46,47,48]. Additional experiments are required to unambiguously determine the mechanism of CV extract action on the LPS-induced TLR4 expression. These experiments may be related to blocking of the TLR4 signalling pathway along with the expression analysis of activated NF-κB.

In conclusion, our results demonstrate that the CV extract promotes cytotoxicity against cancer and endothelial cells in the pro-inflammatory environment. It also has the ability to inhibit the production and expression of the pro-tumorigenic factors associated with inflammation.

## 4. Materials and Methods

### 4.1. Cell Culture

Human umbilical vein endothelial cells (HUVEC) were purchased from Thermo Fisher Scientific (Waltham, MA, USA). The cells were cultured in Medium 200 supplemented with Low Serum Growth Supplement (LSGS), 100 IU/mL penicillin, and 100 µg/mL streptomycin. Cells were cultured in culture flasks coated with Attachment Factor Protein containing gelatine (all compounds from Thermo Fisher Scientific, Waltham, MA, USA). The MCF-7 human breast cancer cell line was obtained from the European Collection of Cell Cultures (Lot. 13K023; Salisbury, UK). The cells were cultured in RPMI 1640 medium containing 2 mM L-glutamine, 10% heat-inactivated foetus bovine serum (FBS), 100 µg/mL streptomycin, and 100 IU/mL penicillin and non-essential amino acids (all compounds from Sigma-Aldrich, Darmstadt, Germany). Both cell lines were maintained at 37 °C in a humidified atmosphere with 5% CO_2_.

### 4.2. Preparation of the CV Extract and LPS Solution

The extract from *Coriolus versicolor* was purchased from commercially available capsules (MycoMedica Company, Police nad Metují, Czech Republic). As stated by the manufacturer, the major soluble components constitute approximately 25% of the CV capsule. The complete culture media were used to dissolve the CV extract as a stock solution of 8 mg/mL and 4 mg/mL, respectively, for 48 h at room temperature with continuous agitation. Insoluble material was removed by centrifugation at 2000 × *g* for 10 min. The soluble supernatant containing 2 mg/mL or 1 mg/mL of the CV extract was sterilized using a 0.22 µm filter, and further diluted with the complete culture media to the defined concentrations. Since a suitable, complete culture medium for the respective cell line was used to dissolve the CV extract, complete Medium 200 for the HUVEC cells and complete RPMI 1640 medium for the MCF-7 cells were used as the vehicle controls.

Lipopolysaccharide (LPS) derived from *Escherichia coli* (0111:B4; Sigma Aldrich, Darmstadt, Germany) was dissolved in PBS and was then added to the suitable cell culture medium to obtain the desired treatment concentrations: 100 ng/mL and 1 µg/mL for HUVEC cells or 2 and 20 µg/mL for MCF-7 cells.

### 4.3. Cell Treatment

Cells were seeded in standard tissue culture plates in a suitable density for each assay and pre-incubated for 24 h. Then, both cell lines were co-stimulated with the CV extract (50, 100, 200, and 300 µg/mL) and LPS (100 ng/mL and 1 µg/mL for HUVEC cell treatment for 24 h or 2 and 20 µg/mL for stimulation of MCF-7 cells for 48 h). The stimulation time and LPS concentration used for the treatment were determined based on conditions that were non-toxic to the cells, and demonstrated simultaneous effective production of a large amount of cytokines. In separate experiments, the cells were treated only with the CV extract or LPS solution. The control cells were incubated alone with complete growth culture medium.

### 4.4. Cell Viability

To determine the level of cell viability, an MTT assay was used to detect the reduction of MTT (3-(4,5-Dimethylthiazolyl)-2,5-diphenyl-tetrazolium bromide; Sigma Aldrich, Darmstadt, Germany) by mitochondrial dehydrogenase to a blue formazan product, which reflects the normal functioning of mitochondria and hence the metabolic rate of the cells. For each experiment, 0.5 × 10^4^ cells were seeded in 96-well tissue culture plates and were pre-incubated for 24 h. Then, different concentrations of CV extract and LPS were added to the cultures. Blank wells contained culture medium with the corresponding CV extract and/or LPS concentrations without cells. Plates were incubated for 24 h (HUVEC cells) or 48 h (MCF-7 cells). After treatment, 100 µL/well of the MTT solution (0.5 mg/mL of MTT reagent in PBS) was added to each well. Culture plates were incubated at 37 °C for 3 h. Subsequently, the formazan product formed by the viable cells was dissolved in 100% DMSO (100 µL/well). The optical density was measured at 570 nm (with reference wavelength of 630 nm) using the Synergy HT Multi-Mode Microplate Reader (BioTek Instruments, Winooski, VT, USA). The results were presented as a percentage of the untreated control cells, which served as 100%.

### 4.5. Cytotoxicity Assay

Cells were seeded and treated as described above. After stimulation, culture cell supernatants (150 μL) were transferred into a new 96-well microplate and the cytotoxicity was assessed using Lactate Dehydrogenase (LDH) Cytotoxicity Detection Kit (ScienCell Research Laboratories, Carlsbad, CA, USA) according to the manufacturer’s instructions. Cells in the positive control wells were treated with a 1% Triton X-100 solution (PanReac AppliChem, Darmstadt, Germany) for 30 min. In the negative control wells, the cells were incubated in culture media alone. Blank wells contained the corresponding CV extract and/or LPS concentrations without cells. The amount of formazan, which is proportional to the amount of LDH release from the dead cells, was measured spectrophotometrically at 490 nm (Synergy HT Multi-Mode Microplate Reader, BioTek Instruments, Winooski, VT, USA). The LDH release level in the treated cells was reported as a percentage of the positive control. All calculations were performed after respective blank absorbance subtraction. Using data from the MTT and LDH assays, the 50% inhibitory concentrations (IC_50_) were determined using GraphPad Prism 7.0 (GraphPad Software Inc., San Diego, CA, USA).

### 4.6. Measurement of ROS Levels

To evaluate the generation of reactive oxygen species (ROS) in the CV extract/LPS-stimulated cells, ROS accumulation was detected using 2′7′-dichlorodihydrofluorescein diacetate (DCFH-DA, Sigma-Aldrich, Darmstadt, Germany). The cells were seeded in 96-well cell culture plates at a density of 2.5 × 10^4^ cells/well and pre-incubated for 24 h. Then, cells were washed twice with PBS and incubated with 20 µM DCFH-DA (200 µL/well) at 37 °C for 30 min in the dark. The DCFH-DA solution was removed, and the cells were washed again with PBS. Subsequently, the cells were treated with various concentrations of CV extract and/or LPS for 24 h (HUVEC cells) or 48 h (MCF-7 cells). Finally, fluorescence was measured in a Synergy HT Multi-Mode Microplate Reader (BioTek Instruments, Winooski, VT, USA) using excitation at 485 nm and emission at 528 nm. Blank wells contained the corresponding CV extract and/or LPS concentrations or media without cells. All calculations were performed after respective blank absorbance subtraction. The results were expressed as a percentage of untreated control cells (served as 100%).

### 4.7. Wound-Healing Assay (Scratch Assay)

The suspensions of cells were seeded in a 12-well plate (0.1 × 10^6^ cells/well) and incubated in the culture medium until the cells reached a 100% confluence. Afterwards, the cell monolayers were mechanically scratched with a 10 μL pipette tip and the unbound cells were removed by washing with PBS. Then, the HUVEC cells were stimulated with a CV extract (100 µg/mL) and/or LPS (100 ng/mL) for 24 h, and the MCF-7 cells were treated with the CV extract (100 µg/mL) and/or LPS (20 µg/mL) for 48 h, respectively. The control cells were cultured in a culture medium without any active agents. Cell migration into the scratched region was recorded using the inverted microscope Leica DMi1 with a digital camera (Wetzlar, Germany). Cell migration at 0 h and 24 h or 48 h was captured, and the wound closure distance was calculated by Image J software (National Institutes of Health, Bethesda, MD, USA). The scratch closure rate is expressed in a percentage by the following formula:

Scratch closure rate (%) = (D0 – Dt)/D0 × 100%

where D0 is the scratch distance at 0 h and Dt is the scratch distance at the designated time.

### 4.8. Cytokine and Matrix Metalloproteinase Assays

Cells were seeded in 24-well tissue culture plates at a density of 0.1 × 10^6^ cells/well and pre-incubated for 24 h. Then, they were simultaneously treated with experimental concentrations of the CV extract and LPS for 24 h (HUVEC cells) or 48 h (MCF-7 cells). In separate experiments, the cells were separately stimulated with the CV extract or LPS. After stimulation, the culture plates were centrifuged at 800 × *g* for 5 min and the culture media were collected and stored at −80 °C. After treatment, the total protein concentration of the viable cell pellets was also determined using a Pierce™ BCA Protein Assay Kit (Thermo Fisher Scientific, Waltham, MA, USA) according to the manufacturer’s instructions. The IL-6, IL-8, and MMP-9 levels were determined by standard ELISA kits from R&D Systems (Minneapolis, MN, USA) according to the manufacturer’s instructions. Colorimetric changes in the assays were detected using the Synergy HT Multi-Mode Microplate Reader (BioTek Instruments, Winooski, VT, USA). Total amounts of the inflammatory factors in the culture media were normalised to the total protein amount of the viable cell pellets. The results are presented as the percentage of LPS-stimulated cells. The concentrations of IL-6, IL-8, and MMP-9 in the culture media are presented in the Supplementary Material (Figure S1).

### 4.9. Western Blot Analysis

Western blot analysis was performed to detect the protein levels of TLR4 and p-IκB in the HUVEC cells stimulated with the CV extract (100 µg/mL) and/or LPS (100 ng/mL) for 24 h, and in the MCF-7 cells treated with the CV extract (100 µg/mL) and/or LPS (20 µg/mL) for 48 h. After treatment, the cells were rinsed with ice-cold PBS and lysed in 70 µL of a buffer containing 4% SDS, 20% glycerol, 4% 2-mercaptoethanol, 0.004% bromophenol blue, 0.250 M Tris HCl (pH 6.8), and 2 mM EDTA. The protein concentration in the lysates was measured using a Pierce™ BCA Protein Assay Kit (Thermo Fisher Scientific, Waltham, MA, USA), according to the manufacturer’s instruction. The lysates were supplemented with sample buffer and subjected to electrophoresis using a 4–20% precast polyacrylamide gel. After transfer onto nitrocellulose, the membranes were immunoblotted with mouse anti-TLR4 IgG (Santa Cruz Biotechnology, Dallas, TX, USA) and rabbit anti-phosphorylated-IκB IgG (Ser32; Cell Signaling, Leiden, The Netherlands) or mouse anti-actin IgG (MP Biomedicals, Santa Ana, CA, USA), followed, as appropriate, by anti-rabbit (Merck Millipore, Burlington, MA, USA) or anti-mouse IgG (Jackson, ImmunoResearch, Cambridge, UK) conjugated with peroxidase. Immunoreactive bands were visualised by chemiluminescence using SuperSignal West Pico substrate (Thermo Fisher Scientific, Waltham, MA, USA) and analysed densitometrically using the ImageJ program (National Institute of Mental Health, Bethesda, MD, USA).

### 4.10. Statistical Analysis

GraphPad Prism 7.0 software (GraphPad Software Inc., San Diego, CA, USA) was used to perform the statistical comparisons between the different values. All values are reported as the mean ± standard error of the mean (SEM) and were analysed using analysis of variance followed by Bonferroni multiple comparisons test with the level of significance set at *p* < 0.05.

## Figures and Tables

**Table 1:** Calculated IC_50_ (µg/mL) values for the human umbilical vein endothelial cells (HUVECs) and MCF-7 cells stimulated with an extract from the fungus *Coriolus versicolor* (CV), with or without different doses of lipopolysaccharide (LPS).

Compounds	MTT Assay IC_50_ (µg/mL)	LDH Assay IC_50_ (µg/mL)
HUVEC Cells		
CV extract	1185 ± 33	895 ± 22
CV extract + 100 ng/mL LPS	746 ± 13	493 ± 9
CV extract + 1 µg/mL LPS	722 ± 15	431 ± 11
MCF-7 Cells		
CV extract	984 ± 24	738 ± 18
CV extract + 2 µg/mL LPS	863 ± 11	624 ± 15
CV extract + 20 µg/mL LPS	770 ± 15	518 ± 12

## Supplementary Materials

The following are available online at https://www.mdpi.com/1422-0067/21/23/9063/s1, Figure S1. Inhibitory effect of the extract from *Coriolus versicolor* on the LPS-induced synthesis of IL-6, IL-8 and MMP-9 (E–F) by human umbilical vein endothelial cells (HUVECs) and MCF-7 human breast cancer cells, respectively.
