# Supplementary material for: Extract from the Coriolus versicolor Fungus as an Anti-Inflammatory Agent with Cytotoxic Properties against Endothelial Cells and Breast Cancer Cells
Source: Int J Mol Sci. 2020 Nov 28;21(23):9063. doi: 10.3390/ijms21239063 (PMC7731170; doi:10.3390/ijms21239063)
Supplement: Supplementary file 1 [file ijms-21-09063-s001.pdf]

Extract from the *Coriolus versicolor* Fungus as an Anti-Inflammatory Agent with Cytotoxic Properties against Endothelial Cells and Breast Cancer Cells

Tomasz Jędrzejewski [1,*], Justyna Sobocińska [1], Małgorzata Pawlikowska [1], Artur Dzialuk [2,*] and Sylwia Wrotek [1]

[1] Department of Immunology, Faculty of Biological and Veterinary Sciences, Nicolaus Copernicus University, 1 Lwowska Str., 87-100 Torun, Poland; j.sobocinska@umk.pl (J.S.); m_pawlikowska@umk.pl (M.P.); wrotek@umk.pl (S.W.)

[2] Department of Genetics, Faculty of Biological Sciences, Kazimierz Wielki University, 10 Powstańców Wielkopolskich Ave., 85-090 Bydgoszcz, Poland

* Correspondence: tomaszj@umk.pl (T.J.); dzialuk@ukw.edu.pl (A.D.)

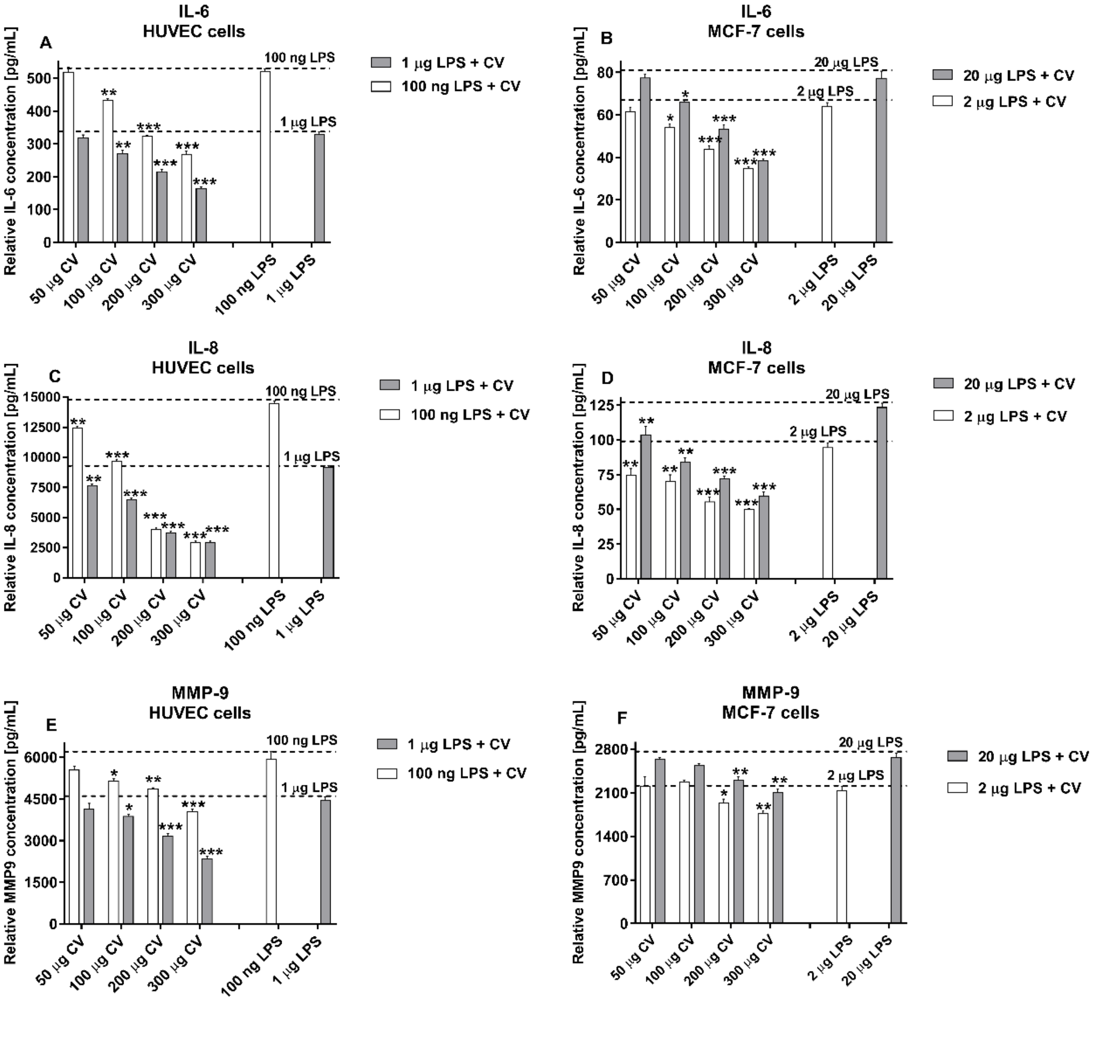

Figure S1. Inhibitory effect of extract from *Coriolus versicolor* (CV) on the LPS-induced synthesis of IL-6 (A-B), IL-8 (C-D) and MMP-9 (E-F) by Human Umbilical Vein Endothelial Cells (HUVEC) and MCF-7 human breast cancer cells, respectively. Data are shown as means ± SEM of five independent experiments. Concentration [pg/ml] of the cytokines and MMP-9 in the culture media were measured by ELISA assays and normalized to the total protein amount of the viable cell pellets. Asterisks denote significant differences between the cells co-

stimulated with CV extract and LPS in comparison with the cells treated with LPS at the given concentration (*P<0.05; **P<0.01; ***P<0.001). The dash lines show the level of factors released from the cells stimulated with LPS at the appropriate concentration.
